# Yellow lupin (*Lupinus luteus* L.) transcriptome sequencing: molecular marker development and comparative studies

**DOI:** 10.1186/1471-2164-13-425

**Published:** 2012-08-24

**Authors:** Lorena B Parra-González, Gabriela A Aravena-Abarzúa, Cristell S Navarro-Navarro, Joshua Udall, Jeff Maughan, Louis M Peterson, Haroldo E Salvo-Garrido, Iván J Maureira-Butler

**Affiliations:** 1 Agriaquaculture Nutritional Genomic Center, CGNA, Genomics and Bioinformatics Unit, Km 10 Camino Cajón-Vilcún, INIA, Temuco, Chile; 2 Plant and Wildlife Science Department, Brigham Young University, 150 E 1230 North, Provo, UT, 84602, USA; 3 Institute of Agricultural Research (INIA), P.O. Box 58-D, Temuco, Chile

**Keywords:** *Lupinus luteus*, EST-SSR, Orphan crop, Microsynteny

## Abstract

**Background:**

Yellow lupin (*Lupinus luteus* L.) is a minor legume crop characterized by its high seed protein content. Although grown in several temperate countries, its orphan condition has limited the generation of genomic tools to aid breeding efforts to improve yield and nutritional quality. In this study, we report the construction of 454-expresed sequence tag (EST) libraries, carried out comparative studies between *L. luteus* and model legume species, developed a comprehensive set of EST-simple sequence repeat (SSR) markers, and validated their utility on diversity studies and transferability to related species.

**Results:**

Two runs of 454 pyrosequencing yielded 205 Mb and 530 Mb of sequence data for L1 (young leaves, buds and flowers) and L2 (immature seeds) EST- libraries. A combined assembly (L1L2) yielded 71,655 contigs with an average contig length of 632 nucleotides. L1L2 contigs were clustered into 55,309 isotigs. 38,200 isotigs translated into proteins and 8,741 of them were full length. Around 57% of *L. luteus* sequences had significant similarity with at least one sequence of *Medicago*, *Lotus*, *Arabidopsis*, or *Glycine*, and 40.17% showed positive matches with all of these species. *L. luteus* isotigs were also screened for the presence of SSR sequences. A total of 2,572 isotigs contained at least one EST-SSR, with a frequency of one SSR per 17.75 kbp. Empirical evaluation of the EST-SSR candidate markers resulted in 222 polymorphic EST-SSRs. Two hundred and fifty four (65.7%) and 113 (30%) SSR primer pairs were able to amplify fragments from *L. hispanicus* and *L. mutabilis* DNA, respectively. Fifty polymorphic EST-SSRs were used to genotype a sample of 64 *L. luteus* accessions. Neighbor-joining distance analysis detected the existence of several clusters among *L. luteus* accessions, strongly suggesting the existence of population subdivisions. However, no clear clustering patterns followed the accession’s origin.

**Conclusion:**

*L. luteus* deep transcriptome sequencing will facilitate the further development of genomic tools and lupin germplasm. Massive sequencing of cDNA libraries will continue to produce raw materials for gene discovery, identification of polymorphisms (SNPs, EST-SSRs, INDELs, etc.) for marker development, anchoring sequences for genome comparisons and putative gene candidates for QTL detection.

## Background

*L. luteus* is a member of the genistoid clade of the Fabaceae family (2n = 52), which is the third largest flowering plant family with over 700 genera and 20,000 species [1]. The genus *Lupinus* comprises more than 200 annual and perennial herbaceous species of which several are cultivated and used as human food or animal feed [2]. Some of them show high levels of tolerance to biotic and abiotic stresses. For instance, *L. hispanicus,* a wild relative of *L. luteus*, has high tolerance to diseases and good adaptation to poor soils, but high levels of bitter alkaloids and low agronomic yields [3]. Lupins are considered to be of polyploid origin which probably played a crucial role in the evolution of their ancestral genomes [4,5]. The major cultivated species are the old world lupin *L. albus* (white lupin), *L. angustifolius* (narrow-leafed lupin), *L. luteus* (yellow lupin), and the new world species *L. mutabilis* (pearl lupin or tarwii) [6].

*L. luteus* is widely distributed across the Mediterranean region, has shallow soil requirements, and cultivated accessions have variable seed yields in Mediterranean environments [7]. In addition, yellow lupin seeds have the highest protein content and twice the cysteine and methionine content of most lupins [8,9]. However, despite its highly nutritional qualities, there is a lack of genetic and molecular tools to aid the genetic breeding of this species.

EST sequencing has accelerated gene discovery when genome sequences are not available, facilitating gene family identification and development of molecular markers. Next-generation sequencing has generated enormous amount of expressed sequence data for a wide number of plant species, specially minor or orphan crops [10]. For example, EST and genome sequencing of lentil and chickpea would not have been feasible without next-generation sequencing [11,12]. The lower cost and greater sequence yield has allowed the identification of candidate genes, even when they are expressed at low levels [13,14].

Research on plants, animals and fungi has shown that sequences of expressed genes are often widely transferable among species, and even genera, allowing wide genome comparative mapping studies [15,16]. For instance, the combination of orphan crop EST sequences with model plant genetic and genomic resources, such as *Lotus japonicus* (Japanese trefoil) and *Medicago truncatula* (barrel medic), has identified macro- and micro-scale synteny, discovered new genes and alleles, and provided insights into genome evolution and duplication [17,18]. Comparisons between ESTs and gene sequences among several legume species have allowed comparative genome studies between *L. albus* and *M. truncatula*[19], and *L. angustifolius* and *Lotus japonicus*[20].

Several molecular markers have been developed for *Lupinus* species, including RFLPs, ITAPs (Intron targeted amplified polymorphic sequences), and AFLPs, which have been used to build genetic linkage maps in *L. albus*[19] and *L. angustifolius*[20,21]. So far, a limited number of SSRs have been developed for *Lupinus* species, and very few of these are EST-SSRs *i.e.* SSRs that are found in expressed sequences [21-23]. Genomic and EST-SSRs have been widely used for the improvement of major crop plants, but their initial development with traditional methods requires significant research investment. Now, an almost unlimited number of genomic and EST-SSRs can be readily developed from next-generation sequencing approaches within most crop species, including orphan crops such as lupin [24-28]. The expressed nature of EST-SSRs allows the annotation of these markers with putative functions by sequence homology and potentially reduces the genetic distance between marker and causal gene to 0 cM. [29,30]. For instance, the length of a dinucleotide SSR at the 5’ UTR of a waxy gene has been associated with amylase content in rice [31,32]. EST-SSRs have also been associated with several disease resistant genes in wheat and rice [33,34] and a number of agronomically important traits in cotton, maize and narrow-leafed lupin [35-37].

In this study, we constructed 454-EST libraries, carried out comparative studies between *L. luteus* and model legume species, and mapped *L. luteus* expressed sequences on the *M. truncatula* chromosomes. Alignments between our putative *L. luteus* genes and their homologs in *M. truncatula*, coupled with amplifications of intergenic regions provided evidence of microscale synteny between both species. In addition, we developed EST-SSR markers and illustrated their utility within diverse accessions of yellow lupin. Finally, because these EST-SSR markers are gene-based, they are also likely conserved among different species of lupin. We evaluated EST-SSR utility in the other *Lupinus* species, *L. mutabilis* and *L. hispanicus.*

## Methods

### Library construction and 454 sequencing

cDNA libraries were constructed from mRNA isolated from two tissue pools. Pool 1 (L1) included young leaves, buds and flowers, and pool 2 (L2), seeds in different developmental stages. RNA from pool 1 and 2 was isolated separately according to the guanidine hydrochloride method [38]. Both RNAs were assessed for quality by inspecting rRNA bands on an Agilent Bioanalyzer (Agilent Technologies, CA, USA).

cDNAs libraries were normalized and prepared using procedures for Roche 454 Titanium sequencing (Roche, Branford, CT, USA). cDNAs from L1 and L2 were synthesized using the stratagene AccuScript High Fidelity RT-PCR System (Agilent Technologies, CA, USA) and 5’ specific adaptors from Clontech. A cDNA normalization was used to improve coding sequence coverage, avoid AT homopolymer artifacts, and reduce excessive 3’ end transcript sequence [39]. cDNAs from both libraries were amplified using the Clontech Advantage HF system (Clontech Laboratories, Inc) and normalized utilizing the Evrogen Trimmer cDNA Normalization kit (Axxora, LLC). These un-cloned, normalized cDNA libraries were prepared for pyrosequencing according to the manufacturers specifications. One 454 run of sequencing was performed for each EST library (454 Life Sciences, Roche).

Separate transcriptome assemblies of L1 and L2 libraries were created using Newbler (*de novo* sequence assembly software of Roche 454 Life Sciences) and the cDNA option. A third assembly (L1L2) was completed using the reads from both libraries to avoid sequence redundancy when developing SSR markers. Reads were initially assembled into contigs and contigs into isotigs, which are equivalent to splice transcriptional variants. Sequence read EST data for L1 and L2 are available through the Sequence Read Archive (SRA055806).

### EST annotation, function and comparative genomics to other species

Comparing isotigs from the combined assembly (L1L2) to the curated non-redundant protein database (nr, http://www.ncbi.nlm.nih.gov; blastx, e value ≤ 1e^-10^) provided a functional annotation for each isotig. Alignments of translated-isotigs and proteins with an e-value ≤ 1e^-40^ were considered to have significant homology. Annotations of the aligned proteins were extrapolated to annotate our putative isotig sequence using Blast2GO (http://www.blast2go.org). To directly compare the lupin isotigs to the genes of other crops, blast searches were also used to compare isotig translations to *Arabidopsis thaliana*, *Glycine max*, *Medicago truncatula* and *Lotus japonicus* Gene Indices (tblastx, e-value ≤ 1e^-10^). Isotigs were also annotated using Gene Ontology (GO) annotations from InterProScan (http://www.ebi.ac.uk).

### *In silico* lupin EST mapping and microsynteny

Blast was used to compare lupin EST isotigs to the Medicago genome 3.0 release (≤ 1e^-20^, HSP identity 60% and HSP length > 50 bp.) The Blast results were visualized using GBrowse where positive matches were displayed as featured tracks on GBrowse 2.13 [40]. The presence of microsynteny was evaluated by PCR amplification of putatively conserved chromosome blocks between *L. luteus* and *M. truncatula*. Where alignments between yellow lupin and *M. truncatula* were identified, specific primer pairs were designed to amplify intergenic regions (Additional file 1). These targeted, intergenic regions were PCR amplified from two *L. luteus* and one *L. hispanicus* accessions using 100 ng of genomic DNA in 20 ul reactions containing 100 ng of genomic DNA, 0.2 mM dNTPs, 2 mM MgCl2, 1X PCR buffer, 2.5% DMSO, 1 U taq polymerase (Agilent Technologies, Santa Clara, CA) and 5 pmoles of each forward-reverse primer pair. PCR reactions were carried out following a touchdown protocol on a peltier thermalcycler (MJ Research, Inc.) 94°C for 5 min; 5 cycles of 1 min at 94°C, 1 min at 55-65°C decreasing 1°C per cycle, 2 min at 72°C followed by 35 cycles of 1 min at 94°C, 1 min at 50-60°C and 2 min at 72°C. Amplicons were purified from agarose gels and sequenced. These amplified, intergenic sequences were mapped onto the *M. truncatula* genome and visualized within a local implementation of GBrowse (Additional file 1). Positive PCR microsynteny set of primers were additionally tested against a screening panel consisting of six diverse accessions of *L. luteus* to search for polymorphisms among yellow lupin genotypes (Additional file 2).

### Identification of EST-SSRs

SSR containing lupin isotigs were identified using the software MISA (MIcroSAtellite, http://www.pgrc.ipk-gatersleben.de/misa). SSR search criteria changed according to repeat types. Di-, and tri-repeats were selected with a minimum length of 12 and 15 nucleotides, respectively. For tetra-, penta- and hexa-repeats, the minimum length was 20 nucleotides. Mononucleotide repeats were not considered due to the possibility of 454 homopolymer sequencing errors associated with this technology. To estimate the amount of SSRs included in coding regions, L1L2 sequences were analyzed using ESTScan (http://www.ch.embnet.org/software/ESTScan.html). ORFs discovery was carried out using default parameters and putative cd sequences scanned for SSR motifs using MISA.

From all selected-SSR containing isotigs, only sequences with a motif of at least 7 repeat units were considered for primer design. Flanking primer pairs were designed using the Primer3 software available at NCBI v.3.12 with expected amplicon lengths between 150 - 500 bp. Oligonucleotides were synthesized by IDT (Integrated DNA Technologies, Inc.).

### Evaluation and utility of EST-SSRs

EST-SSR polymorphisms and transferability were evaluated on the germplasm screening panel previously mentioned*,* and one accession each of *L. hispanicus* and *L. mutabilis*.

DNAs were extracted following standard procedures [41], quantified using a synergy HT Multimode Microplate Reader (Biotek Instruments, Winooski, VT), and diluted to 50 ng/ul in TE buffer (10 Mm TRIS, 1 mM EDTA pH 7.5). DNA amplification was carried out in 20ul PCR reactions as described above.

PCR products were separated on 6% denaturing polyacrylamide gels, run in TBE buffer at 60 watts for 3–4 hours and visualized using silver stain procedures. DNA amplicons of six EST-SSR primer-pairs used in the polymorphism screening were purified from agarose gels and sequenced in an Applied Biosystems 3730xl DNA Analyzer sequencer (Applied Biosystems, Carlsbad, CA). Amplicon sequences from each EST-SSR primer-pairs were aligned using Geneious version 5.5.3.0 (Biomatters Ltd., using default parameters).

### Genetic diversity

The polymorphic EST-SSRs were evaluated in sixty-four *L. luteus* accessions from several origins (Poland, Ukraine, the former Soviet Union, Spain, Germany, Morocco, Belarus, Portugal, Netherlands, Israel, Hungary, and Chile; Additional file 2). Polish accessions were kindly provided by W.K. Swiecicki, Institute of Plant Genetics, Polish Academy of Sciences, Poznan. Our collection of Chilean accessions is composed of improved breeding lines that are adapted to the Chilean environment. This Chilean germplasm originated from breeding and selection of old European varieties for Southern Chilean environmental conditions. The rest were obtained from the western Regional PI Station, USDA, ARS, WRPIS, Washington State University, Regional Plant Introduction Station, Pullman, Washington, USA. A sample of 50 polymorphic EST-SSRs was used to genotype the sixty-four *L. luteus* accessions (Table 1). Eighteen EST-SSRs were identified from isotigs specific to L2, 25 isotigs specific to L1, and seven were common to both L1 and L2 libraries. EST-SSR fragments with different sizes were scored as different alleles and coded with alphabetical letters for each primer set. Genetic relationships among *L. luteus* accessions were evaluated using the neighbor-joining algorithm implemented in PAUP* (v4.01b10). A distance tree was built and branch support estimated by 10,000 bootstraps.

**Table 1:** **Characteristics of 50 EST-SSR primers developed in* L. luteus.* Shown for each primer pair are the library specificity, repeat motif, forward and reverse sequence, allele range size (bp), number of alleles, amplification in other Lupin species, and annotation**

**Marker name**	**Library**	**Repeat motif**	**Forward primer (5**^**′**^**-3**^**′**^**)**	**Reverse primer (5**^**′**^**-3**^**′**^**)**	**Size (bp)**	**No of alleles**	**Amplification**	**Annotation**
l1l2itg33000	L1	(ACA)7	CACGTCAGTCCTTGCACCTA	GCACAGCAACAACAACACAA	129-132	2	*L.hispanicus*	
l1l2itg51784	L1	(TA)8	CATCCTTCAAAAACCATTTCAA	AATGTTGATGAACGCGTGTG	274-280	3		
l1l2itg52347	L1	(AT)8	CTCATGTTTCTTGGGTGGAAA	CAATCATGTCTAAACCGGGAA	209-215	4		
l1l2itg50343	L1	(AT)10	ATATTAGCGGCCATGCTGTT	TGTTCATGTTGGTTGCAAGA	235-239	3		
l1l2itg20858	L1	(AAC)12	ACCCCACTTCTCCCAACTCT	TCCATGAATGAAATGGGGTT	229-238	3	*L.hispanicus*	Pollen-specific protein SF3
l1l2itg20038	L1	(TA)9	TTCAGAAACAAAGGGGTTGC	TCCAGAAATTCTTCTACATCCCA	179-183	3		
l1l2itg52625	L1	(TCA)12	CTGGTCTTCTGTCGACTCCA	GACCAAGAAGTCAAGCTCGG	109-124	4		
l1l2itg37631	L1	(CT)12	TAAAGTGCCACCAACAAGCA	TTGTGTTGGTTGTGTGTAGAGAGA	133-155	6		
l1l2itg27097	L1	(AAT)7	TTCAACTACCGGTTGAACCAC	GCCCAGAATTAGGGTGCTTT	206-209	2		
l1l2itg22424	L1	(GAA)7	AAACGACCAACCGCATAAAG	GATGCGTGAAACTGCAAAGA	240-249	3	*L.hispanicus*	N-acetylglutamate synthase
l1l2itg29703	L1	(GA)8	ACCTTTGCGCCAAGATACAC	ATTGTGACGGTTTCACTCCC	213-219	4		
l1l2itg28437	L1	(TA)9	GGGCACATTTGACTCTTTCG	TCCGTGCAATGTCAATATCAA	260-268	4	*L.hispanicus*	
l1l2itg36804	L1	(ATA)12	CACATGAGAAGCAGCAATGAA	ATGCGGTGGAGTGGAAGTAA	254-260	2	*L.hispanicus*	
l1l2itg21177	L1, L2	(CAT)8	CCTTGAGGCCAATAAATGGA	TTAAGGAAGCTAGGGCCACA	217-226	3	*L.hispanicus*	Delta-8 sphingolipid desaturase
l1l2itg39645	L1	(ATT)10	AATCATGGCCTTTTTGCTTG	CGTCTTGCTCTGGTTCTTCC	148-169	5		
l1l2itg35309	L1	(TA)8	TTCATGGCAAGAAAAACATCT	AATCATCCATGCCATTTAACA	271-281	4		
l1l2itg56943	L1	(GA)8	GAGGCCCAAAAACAGAAACA	CCATTTGCGTTCGGTTCTAT	270-272	2	*L.hispanicus, L.mutabilis*	
l1l2itg31693	L1	(TAT)8	AGGGGCAAAGCTCAAAGACT	CATTCACATTTTATCCTCATTGACTC	196-217	4	*L.hispanicus*	
l1l2itg10347	L1	(AT)8	TGTGGTAAATGCAGGCTCAG	ATGCAACGGGAACCATAGTC	184-186	2	*L.hispanicus*	
l1l2itg14618	L1	(CAT)7	TTCCTCATCTCCCACACCTC	AGCTTCTGCTTGTAATCGGC	237-252	4		
l1l2itg20466	L1, L2	(TA)9	GTAATCATTCATGTATAATTGTAACACTC	CAATTCATTATCTGTATTATTACCCC	180-186	3		Cytochrome B561
l1l2itg53474	L1, L2	(GA)10	CTGAAGTGAGGTTCGGGAAG	TCAATCACACATGCTTGTTCC	230-234	3		Cullin-1
l1l2itg51894	L1	(AT)10	TGACTTTGATTGTTTAGCTTACAGG	TGAATGTCAAATGCAATATTAAGGA	247-263	3	*L.hispanicus*	
l1l2itg24819	L1	(AT)8	CATTCATTCTCTAATCTTTTGTGTCA	TAAAGCTTGTCTCTTGCCCG	219-244	5	*L.hispanicus*	
l1l2itg55310	L1	(TA)9	ACCAAAAGGGTGGGTGAAAT	CCTAACATTTGAACATATTTAAAACAA	277-283	4		
l1l2itg14694	L1, L2	(TA)8	AAGTAGGAAGATCGAATATGAACG	GGGAAAATATCGAGGTTTTCATC	268-278	3	*L.hispanicus, L.mutabilis*	RNA-binding protein
l1l2itg35641	L1	(AT)8	AGTTGCAATTCAACAACGCA	CATGCTCTATGGCAAGTGCT	247-251	3		
l1l2itg38340	L1	(TAT)7	AGCTCCACTTTTAGAATTGCG	TCTATTGTTACATGCACATTATCCC	164-173	4	*L.hispanicus*	
l1l2itg26293	L2	(TCCGAA)15	CCTGCAGTGGTAGAACCTGG	GAAGCAAGGTCCACAGAAGG	123-183	6		18S ribosomal RNA gene
l1l2itg42878	L2	(CATTCC)11	CAACTCTTGTTTGCAGACCG	GCTACCCTTTCGGGACTAGC	217-235	4	*L.hispanicus, L.mutabilis*	
l1l2itg13749	L2	(TTCCGC)8	TTTTTACTCGACTCGCTCCC	CCAGTCGATTTAGCAGTCGC	207-261	7	*L.hispanicus, L.mutabilis*	
l1l2itg32760	L1, L2	(CGGAAT)14	TCATAATGAATTAAATTAACCCCC	TCCCTGACTCTGTCTTTGGG	146-284	14	*L.hispanicus*	
l1l2itg00675	L2	(TCT)8(TCG)5	AGAGAGATCCTCTTTGACGCC	GTGGTTAGCGAGAACCATCG	187-199	4		BSD domain-containing protein
l1l2itg45631	L2	(ATC)10	AAACCGAATTGTGGATCAGC	GGGGACTCTGGAAAATCAGG	146-155	3	*L.hispanicus, L.mutabilis*	Alphavirus core protein family
l1l2itg20349	L2	(AAC)7	ACTAAGGGAAAGGGATTCGG	CCAGGCAAGAACAAAAGAGG	186-189	2	*L.hispanicus, L.mutabilis*	LPA2 (low psii accumulation2)
l1l2itg41827	L2	(TTG)7	TTGAGTCATATCACCATAGCGG	CAACCACAAATGGAAAACCC	242-245	2	*L.hispanicus, L.mutabilis*	Lipase class 3 family protein
l1l2itg47916	L2	(TCT)9	GGTGGGTGAAAATGAAATGG	TAACCAAAATGGTTCGTCGG	241-247	2	*L.hispanicus, L.mutabilis*	
l1l2itg42002	L2	(AAC)8	CTTGCAGGGTCTTCTTACAGC	GGGGTTGTTTTTGGTGTCC	243-246	2	*L.hispanicus*	
l1l2itg54849	L2	(ACA)7	TTCTCCAATGATGAAATGCC	TTCACGGCTAAATACCAAGC	177-183	2	*L.hispanicus*	Microtubule-associated protein
l1l2itg13638	L2	(TGT)9	CCATGGTCATCATTAACCCC	CGAGTCGAGTTCGTTTACCC	188-200	5	*L.hispanicus, L.mutabilis*	f-box family protein
l1l2itg26640	L2	(AG)7	GGTCTGTTGGAGAAGGCTACC	CCACCAATGGGTAGACATACG	203-209	3	*L.hispanicus*	Small nuclear ribonucleoprotein
l1l2itg29887	L2	(GCT)10	CCCATCTGAAAGACTTACGGC	TCCCTTTTCATCCAGAGAGG	243-249	2	*L.hispanicus*	Ser/thr-protein kinase AFC2
l1l2itg50945	L2	(CCA)6(ACA)7	CCAGAACAAGGAGAAGGTTCC	TTCTTCTTCCTCGCAGGC	198-204	3	*L.hispanicus*	Zinc finger, Transcription factor
l1l2itg44905	L2	(CTT)9	AAATCACAGAGCCAAGGAGG	TCAGCTTATTTTGTTTCCAAGC	356-362	3	*L.hispanicus, L.mutabilis*	Transcription factor
l1l2itg09113	L2	(AT)8	CATGACCCAATCTCAAACCC	GCATCTGGATCTGCTTAATTGG	341-343	2	*L.hispanicus*	
l1l2itg03938	L2	(CCGATT)9	CATGTGGGAAGACCAGAAGC	ACTACGCGCTGCTAATGTCC	212-290	7	*L.hispanicus, L.mutabilis*	Polygalacturonase
l1l2itg32421	L2	(AATCGG)8	AGAGAAGTAGGCATGGTGGC	GATCGGCCTATTCACTCAGC	221-293	5	*L.hispanicus, L.mutabilis*	
l1l2itg29217	L1, L2	(AT)7	ACACTCTCAAGGAAAAGGGC	CCATTTAACCGATAATGCTTGG	340-344	2	*L.hispanicus*	Lactoylglutathione lyase
l1l2itg27515	L2	(TTC)17	CATGCGTCCAATCTATCACC	AGTGGGAAACAAGGAAGTGG	182-221	8	*L.hispanicus, L.mutabilis*	PPR-containing protein
l1l2itg41211	L2	(GAA)11	TCCTCCTGCTTCAGAACG	AAATCCACGTCATCAATCCG	209-230	6	*L.hispanicus, L.mutabilis*	

## Results

### Seed and leaf-flower EST libraries

Two runs of 454 pyrosequencing yielded 205 Mb and 530 Mb of sequence data for L1 and L2 EST libraries, respectively (Table 2). L1 produced 604,869 usable reads that assembled into 26,975 contigs with an average length of 468 nucleotides. L2 generated 1,345,892 usable reads that assembled into 43,674 contigs with an average length of 800 nucleotides. Careful inspection of the L1 contigs found lower percentages of coding regions, higher A/T content, and 2x more A/T homopolymers than L2 contigs. A combined assembly (L1L2) was created to identify the genes that were common in both tissues. 1,964,517 reads were used in the L1L2 assembly and they formed 71,655 contigs with an average contig length of 632 nucleotides. To reduce sequence redundancy due to transcript and alternative splice variants, L1L2 contigs were clustered into 55,309 isotigs, of which 38,200 isotigs translated into proteins and 8,741 of them were full length.

**Table 2:** **cDNA 454 assembly statistics of L1, L2 and L1L2* L. luteus* libraries**

**Library statistics**	***L.luteus *EST-library**		
	**L1**	**L2**	**L1L2**
Number of sequenced bases	205,618,165	530,678,975	736,297,140
Number of reads	755,206	1,468,202	2,213,408
Number of reads assembled	604,869	1,345,892	1,964,517
Read average length	276	361	332
Number of contigs	26,975	43,674	71,655
Contig average size	589	986	901
Number of isotigs	21,235	35,191	55,309
Isotig average size	589	986	901
Number of isogroups	15,295	24,653	36,886
Isogroup average size	589	989	905
Average number of reads by contig	22.4	30.8	27.4
%GC	30.7	39.9	37.5
Annotated sequences			32,862
Gbrowse mapped sequences			25,400

### Functional classification and *in silico* comparative genomics

**Figure 1:**
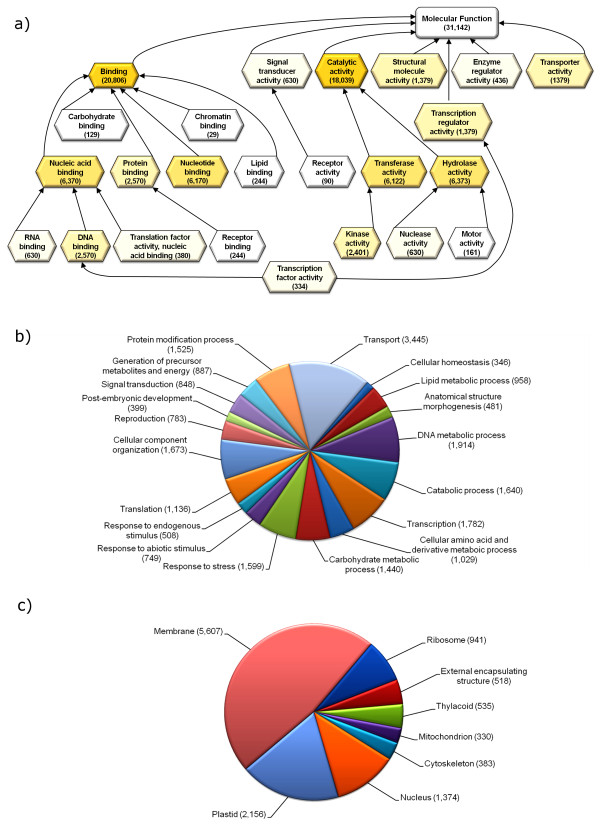
**GO term annotations for L1L2. Isotigs were grouped under three categories: (a) molecular function, (b) biological processes, and (c) cellular components.** Numbers between parentheses indicate the number of positive matches for each function.

The assembled 454 isotigs represented putative transcriptional products *i.e.* functional genes. Blastx was used to annotate the L1L2 putative genes (*i.e.* isotigs). A total of 32,862 (59.5%) putative genes showed matches with other species (≤1e^-10^). Of these sequences, 20,169 (36.5%) showed high similarity to other plant species genes (≤1e^-40^). GO annotations were grouped under three categories: molecular function, biological processes, and cellular components (Figure 1). At least 31,142 isotigs were annotated with one molecular function, 11,894 with a cellular component and 22,842 with biological process.

**Figure 2:**
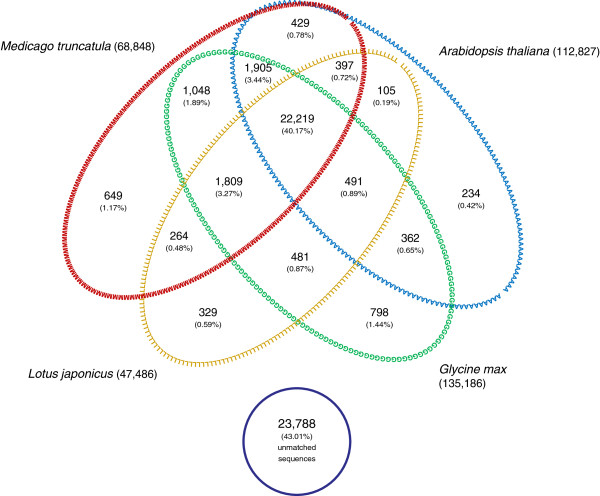
**Venn diagram summarizing the distribution of tBlastX matches between *L. luteus *and four model species (*A. thaliana, M. truncatula, L. japonicus and G. max*).** Numbers following the model species correspond to the size of the respective data base. Numbers within the Venn diagram indicate the number of sequences sharing similarity using tBLASTx. Numbers within parenthesis indicate the percentage of matches in terms of the total number of *L. luteus* sequences.

Blast was used to compare L1L2 to several model species (tblastx; ≤ 1e^-10^; Figure 2). Around 57% (31,520) of *L. luteus* sequences had significant similarity with at least one sequence of *Medicago*, *Lotus*, *Arabidopsis*, or *Glycine*, and 40.17% showed positive matches with all of these species.

### *In silico* mapping of lupin ESTs on *M. Truncatula* chromosomes

Alignment of *L. luteus* isotig sequences to the *M. truncatula* genome (Blastn; ≤1e^-20^; MT3) was used to identify local genomic variability between our ESTs and a related, well-annotated reference genome sequence. The alignments were visualized using GBrowse (v. 2.13) with the Blast matches displayed as feature tracks. A total of 25,400 sequences (46%) from L1L2 had a positive match with MT3 and were distributed heterogeneously on the *M. truncatula* chromosomes. Chromosomes 3 and 1 had the highest (34,636) and lowest (16,055) number of matches, respectively. Each *L. luteus* sequence was mapped to an average of 3.7 positions on the *Medicago* genome.

**Figure 3:**
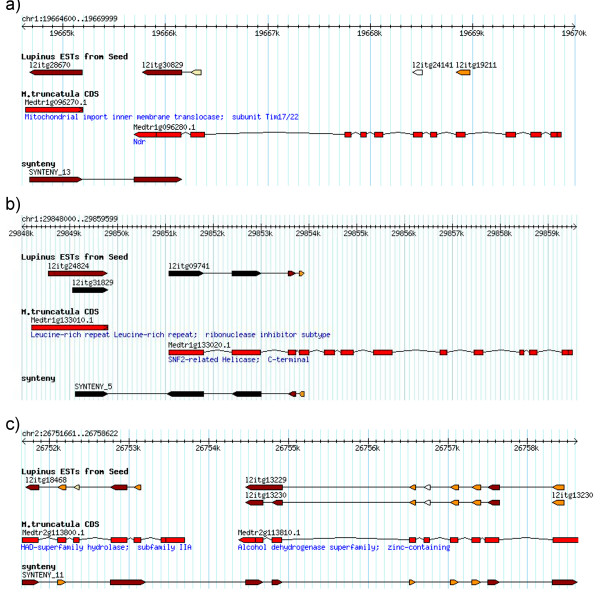
**Microsyntenic *L. luteus *DNA fragments mapped on the Medicago genome using a GBrowse platform.** (**a**) *L. luteus* microsyntenic region 13 on *M. truncatula* chromosome 1; (**b**) *L. luteus* microsyntenic region 5 on *M. truncatula* chromosome 1; (**c**) *L. luteus* microsyntenic region 11 on *M. truncatula* chromosome 2.

Occasionally, independent alignments of lupin genes with the *M. truncatula* genome were found relatively close to each other that primers could be designed to hybridize conserved exons, allowing the amplification of intergenic sequences in between lupin and *M. truncatula* coding sequences (Figure 3). Positive PCR amplification of intergenic regions using *L. luteus* genomic DNA and primers anchored on conserved exonic regions of adjacent *M. truncatula* genes suggested the occurrence of microsynteny (*i.e.* conserved gene order) between yellow lupin and *Medicago.* Thirty-three out of 79 (42%) primer pairs amplified clear PCR products. 16 pairs showed expected sizes based on *Medicago* genomic regions. The remainder primer pairs amplified shorter or longer lupin fragments than the fragments amplified in *M. truncatula*. Amplicon sequence data for *L. luteus* containing intergenic DNA sequence were mapped onto the *Medicago* genome using blast (Figure 3). The alignments between *L. luteus* and *Medicago* showed high levels of conservation in the coding regions, but little sequence similarity in the intergenic regions. When *L. hispanicus* DNA was included as PCR template, only 23 primer pairs amplified. Variable amplification was likely due to localized sequence polymorphism within the primer binding site (*i.e.* small indels) and not the lack of microsynteny. This ratio (23/33) is similar to the number of EST-SSRs that were found to amplify fragments in both species. Alignments among *L. luteus* and *L. hispanicus* were possible at intergenic regions but sequences were clearly less similar than coding regions.

When these markers were evaluated on the screening panel of diverse germplasm accessions, 10 had length polymorphism for these intergenic regions (Additional file 1). In addition to EST-SSRs, this new Conserved Microsynteny (CMS) marker could be valuable resource for crop improvement with molecular markers.

### Identification of EST-SSRs

A total of 2,572 isotig sequences contained at least one EST-SSR, with a frequency of one SSR per 17.75 kilobases (Table 3). The observed frequencies for di-, tri-, tetra-, penta-, and hexa-repeats were 30.4%, 52.7%, 2.4%, 7.5% and 6.2%, respectively (Table 4). Among the di-nucleotide repeats, the AT/TA motif was the most frequently observed (49%) followed by GA/CT (45%). The AC/GT motif was found in low frequency (6%) and there were no CG/GC motifs in the *Lupinus* sequences. Tri-nucleotide repeats, predominantly A/T-rich motifs (74.5%), were the most frequent tri-nucleotide repeat found in the *Lupinus* transcriptome. These tri-nucleotide repeats were often found within the coding sequence of putative genes (77.2%). GAA/CTT motif was the most frequent tri-nucleotide repeat (31%).

**Table 3:** **Features of EST-SSRs identified in assembled L1L2* L. luteus* library**

Total number of examined sequences	55,309
Estimated transcriptome screened (kbp)	49,841
Number of sequences containing SSRs	2,572
Number of identified SSR	2,774
Number of EST-SSRs in coding regions	1,435
Number of sequences containing more than 1 SSRs	147
Number of SSRs present in compound formation	195
Frequency of SSR in transcriptome	1/18 Kbp

**Table 4:** **Distribution of repeat types and number of repeats within the L1L2* L. luteus* library**

**Repeat type**	**Number of repeat units**							**Total (%)**
	**4**	**5**	**6**	**7**	**8**	**9**	**> 10**	
Di-nucleotide			363	204	120	72	91	851 (30.7)
Tri-nucleotide		826	369	131	69	25	57	1477 (53.2)
Tetra-nucleotide		43	9	3	1	2	8	66 (2.4)
Penta-nucleotide	129	46	6	3	9	6	12	209 (7.5)
Hexa-nucleotide	105	26	11	3	9	5	13	171 (6.2)

### Evaluation of EST-SSRs within yellow lupin and other lupin species

**Figure 4:**
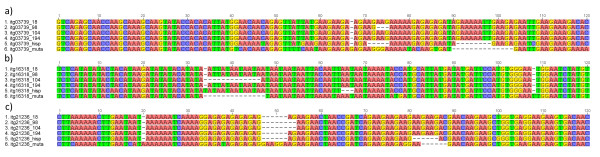
**Alignment of *L. luteus*, *L. hispanicus* and *L. mutabilis *containing several repeat motifs.** (**a**) isotig03739 with GA and AGA motifs; (**b**) isotig16318 with a TAA motif; and (**c**) isotig21236 with a GAA motif.

Studies involving repeat sizes and level of polymorphism have suggested a positive correlation between repeat number and rates of polymorphisms, especially in dimeric microsatellites [28,42]. Thus, only EST-SSRs containing at least 7 repeat units were selected for validation to increase the likelihood of finding markers polymorphic between lupin accessions. A total of 783 EST-SSR candidate loci had sufficient repeat units, but only 375 had enough repeat flanking sequence to be suitable for primer design. PCR amplification of these markers resulted in 222 EST-SSRs (59%) that were polymorphic among the six diverse *L. luteus* included in screening panel. 130 EST-SSRs were monomorphic and 23 primer-pairs failed to amplify. A small number (6) of EST-SSRs were validated by Sanger sequencing. The amplicon sequences from four different *L. luteus* genotypes and from *L. hispanicus* and *L. mutabilis* confirmed the existence of SSR motifs and their length variability between lupin accessions (Figure 4). EST-SSR amplicons showed high conservation at the flanking SSR regions of both *Lupinus* species when compared with *L. luteus*. However, several indels were observed in adjacent regions and within the SSR motif, especially in *L. mutabilis*. 

**Figure 5:**
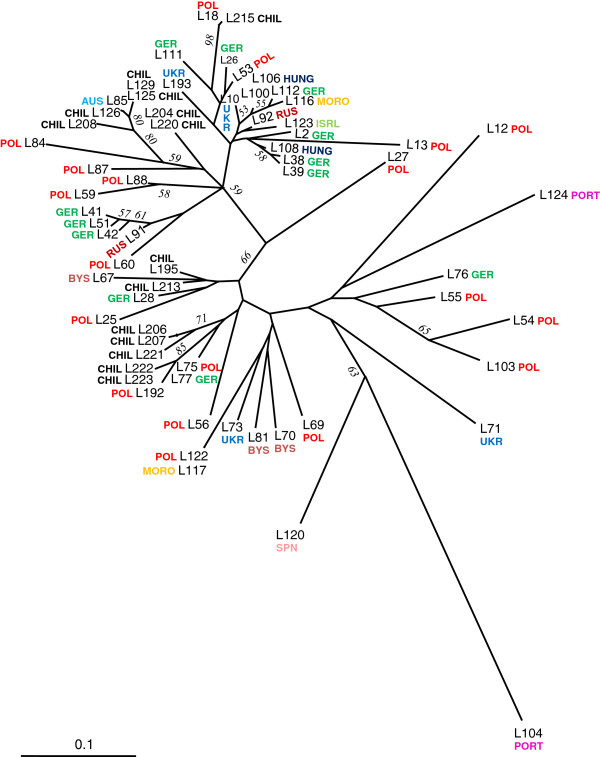
**Neighbour Joining tree relating the 64**  ***L. luteus *accessions included in the diversity study.** Numbers above branches correspond to bootstrap values. Accessions are identified by a letter L followed by numbers. Letters around accessions identify country of origin based on seed bank or breeding histories (RUS: Russia, ISRL: Israel, HUNG: Hungary, CHIL: Chile, GER: Germany, SPN: Spain, PORT: Portugal, MORO: Morocco, POL: Poland, BYS: Belarus, UKR: Ukraine). The scale is in distance units.

Fifty polymorphic EST-SSRs were used to genotype a sample of 64 *L. luteus* accessions (Table 1 and Additional file 2). Twenty-four of these selected markers were specific to L1 (leaf-flower EST library), 20 EST-SSRs were specific to L2 (seed EST library), and 6 were present in both libraries. Neighbor-joining distance analysis detected several clusters among *L. luteus* accessions, strongly suggesting the existence of population subdivisions (Figure 5). However, no clear geographical patterns (country of origin) were observed among lupin accessions. Interestingly, Chilean accessions were distributed in most clusters, probably reflecting the breeding history of these genotypes. Two hundred and fifty four (65.7%) and 113 (30%) SSR primer pairs were able to amplify fragments from *L. hispanicus* and *L. mutabilis* DNA, respectively.

## Discussion

Next-generation sequencing has reduced the existing gap between major crop genomic platforms and the limited resources that are currently available for orphan crops [10]. Complete transcriptome sequencing has generated species specific molecular markers, *in silico* expression analyses, gene discovery, and phylogenetic relationships [43,44].

In this research, we used 454 cDNA sequences to assemble transcriptomes of two tissues (L1 and L2) of yellow lupin. We recovered a large number of previously unknown and uncharacterized yellow lupin gene sequences (Table 2). The total number of sequences for the combined library was mostly additive from L1 and L2. The L1 library favored the inclusion of longer 3’UTR regions, and thus, reducing the amount of coding sequences needed to assemble longer combined contigs (L1L2). As a consequence, two or more sequences belonging to the same transcript may not be assembled together, causing an overestimation of expressed sequences. The larger amount of 3’UTR regions for L1 is also in agreement with the lower GC content, condition typically associated with untranslated regions [45,46]. Undoubtedly, a number of expressed sequences are tissue specific and will not assemble into combined contigs. For instance, several genes related to seed dormancy and germination are not expressed in vegetative and floral tissues [47,48]. The same specificity was observed in a number of tissues and plant species [49-51]. The assembly of L1L2 generated 55,309 isotigs of which 30,811 had similarity to putative proteins found in other plant species. Comparative studies carried out against *L. japonicus, M. truncatula* and *G. max* showed a total of 31,520 lupin sequences similar to at least one of the model legume databases and 22,219 were similar to all of them. *Lotus* and *Medicago* belong to the Galegoid subclade, which includes mostly temperate legume species [52]. *Glycine* is a member of the Phaseoloid subclade which comprises mostly tropical species [52]. Lupins belong to the Genistoid subclade, which is sister (and distant) to most of the described Papilionoid subclades; especially those containing most domesticated species [53].

Although micro-repeat motifs are frequent in plant genomes and their respective transcriptomes, the frequency of SSR discovery depends on the search criteria [42,54-56]. We analyzed 55,309 lupin isotig sequences using MISA and identified 2,796 SSR motifs with an average frequency of one SSR per 17.75 kbp. Tri-nucleotide repeats were the motifs most frequently found in *L. luteus* expressed sequences. Similar results have been reported in numerous plant species [26,28,54,55,57]. The abundance of trimeric EST-SSRs has been attributed to the absence of frameshift mutations when there is length variation in these SSRs [58]. Indeed, 1,435 EST-SSRs were discovered within coding regions of the gene. Among tri-nucleotide repeats, AT-rich motifs were the most predominant ones (74.5%), which have also been observed in soybean, Citrus and Arabidopsis [54,57]. For di-nucleotide repeats, AT was the most frequently observed motif, contrasting with results from Arabidopsis, soybean, maize, rice, wheat and barley where AC/GT were the most frequent repeats [26,28,54,55,57]. The high proportion of untranslated sequences (specifically 3’UTR), mainly contributed from the L1, could explain the bias toward A/T-rich repeat sequences observed in yellow lupin. There were no CG repeats in the lupin sequences, similar to results obtained in barrel medic [24], rice, corn, soybean [57], wheat [27], Sorghum [25], Arabidopsis, apricot and peach [59].

We used GBrowse to visualize lupin ESTs aligned to the *M. truncatula* chromosomes (Figure 3). This approach potentially identifies paralogs sequences and allows color-coded alignment by BLAST significance [60]. A total of 25,400 *L. luteus* contigs were localized and found to be distributed across the entire Medicago genome with chromosomes Mt1 and Mt3 having the highest number of gene matches. Each yellow lupin sequence was mapped to an average of 3.7 locations, which may correspond in part to rounds of genome duplications previously described for the Medicago genome [61]. Understanding syntenic relationships among species is essential to exploit the available tools developed for comparative genomic analysis. Using this approach, we created a new method of developing molecular markers, markers that are based on conserved microsynteny (CMS) between orphan and model species. Genome comparisons among *M. truncatula*, *G. max* and *L. japonicus* have shown that, in general, most genes in Papilionoid legume species are likely to be found within a relatively long syntenic region of any other Papilioniod species [62]. Positive amplification and sequencing of *L. luteus* intergenic regions, based on PCR primers located on *M.* t*runcatula* adjacent genes, suggested the existence of microscale synteny between these legume species. Roughly 40% of the targeted intergenic *L. luteus* regions amplified, points out the usefulness of conserved legume chromosome blocks for genomic studies of orphan crops. Although some primer pairs failed to amplify, poor amplification could be a consequence of non-synteny, but also other technical limitations could also explain negative PCR results. For instance it is known that non-coding DNA regions are highly variable among species [63,64], and negative PCR amplifications could easily due to excessively long *L. luteus* intergenic regions.

Few studies have reported the use of EST-SSRs in *Lupinus* species [19,21,22]. Most efforts have focused on genetic linkage mapping and in diversity studies in *L. angustifolius*[20], *L. albus*[21] and *L. luteus*[22]*.* To validate our *L. luteus* polymorphic markers we tested 50 EST-SSRs on a population of 64 genotypes of *L. luteus*. An analysis of genotypic diversity illustrated the existence of several clusters within *L. luteus* germplasm. The lack of a clear pattern following the geographical accession origin (country) could be explained by three reasons. 1) The number of accessions may not have been large enough to allow a clear pattern to emerge. 2) *L. luteus* is widely distributed across the Mediterranean region, mainly due to human introductions [6]. This situation could have homogenized natural genetic distinctiveness, leaving mostly population subdivisions based on breeding histories. 3) Finally, it is possible some accessions could have been misclassified; and thus, obscuring an existing geographical clustering pattern.

We observed that a number of high yellow lupin EST-SSR amplified fragments in two other lupin species, *L. hispanicus* and *L. mutabilis* (Table 1). The high number of transferable markers between *L. luteus* and *L. hispanicus* confirmed their closer genetic relationship [5,65] than *L. luteus and L. mutabilis*. The two closely related species have the same chromosome number (2n = 52) and are still interfertile, generating a natural hybrid called *hispanicoluteus*[66]. Phylogenetic studies have placed new and old world lupins into two different clades [5,65,67]. Thus, most EST-SSRs amplified in *L. mutabilis* (2n = 48), the only cultivated new world lupin [65], should have high transferability rates to other lupin species, such as *L. albus* and *L. angustifolius.* The understanding of the genetic diversity among other close relative lupin species will facilitate the transfer of favorable variation into cultivated species. For instance, *L. hispanicus* has been suggested as a reservoir of favorable variation for a number of biotic and abiotic stresses currently affecting *L. luteus*[68,69].

## Conclusion

*L. luteus* deep transcriptome sequencing will facilitate the further development of genomic tools and lupin germplasm. Massive sequencing of cDNA libraries will continue to produce raw materials for gene discoveries, identification of polymorphisms (SNPs, EST-SSRs, INDELs, etc.) for marker development, anchoring sequences for genome comparison studies and putative gene candidates for QTL detection. We are also exploiting the microsyntenic regions observed among *L. luteus* and legume model species to saturate yellow lupin linkage maps by amplifying conserved regions across legume species. The utilization of these tools will allow transforming *L. luteus* into a valid temperate legume crop alternative.

## Competing interests

The authors declare that they have no competing interests.

## Authors’ contributions

LBP collected the tissues and extracted the RNAs. JU, LBP and JM constructed the EST libraries. JU and JM supervised the 454 sequencing of the libraries. LBP and JU conducted the SSR search and primer design. LBP conducted the SSR polymorphism tests and transferability studies. LMP sequenced the amplicons of SSR and intergenic blocks. GAA grew the plants for the diversity study, extracted the DNAs, PCR amplified and conducted the genotyping of the population. IMB drafted the experimental design of all the studies carried out in this work and conducted the genetic analysis for the diversity study. CSN conducted annotations and *in silico* mapping of the sequences. LBP conducted the microscale synteny studies. HS and IMB conceived the study. LBP drafted the manuscript with the support of IMB. All the authors read and approved the final manuscript.

## Supplementary Material

Additional file 1 ** Table S1.** Characteristics of 33 Conserved Microsynteny (CMS) markers developed in *L. luteus.* Shown for each primer pair are the Medicago chromosome library specificity, l1l2 isotigs where CMS forward and reverse primers were anchored, forward and reverse sequence, expected Medicago amplicon size (bp), *L. luteus* CMS amplicon size (bp), amplification in other Lupin species (*L. hispanicus*), and the level of polymorphism on the *L. luteus* screening panel. Click here for file

Additional file 2 ** Table S2. ***Lupinus luteus*, *L. hispanicus* and *L. mutabilis* accessions included in the study. Click here for file
