# Supplementary material for: Yellow lupin (Lupinus luteus L.) transcriptome sequencing: molecular marker development and comparative studies
Source: BMC Genomics. 2012 Aug 24;13:425. doi: 10.1186/1471-2164-13-425 (PMC3472298; doi:10.1186/1471-2164-13-425)
Supplement: Additional file 1 — Table S1. Characteristics of 33 Conserved Microsynteny (CMS) markers developed in L. luteus. Shown for each primer pair are the Medicago chromosome library specificity, l1l2 isotigs where CMS forward and reverse primers were anchored, forward and reverse sequence, expected Medicago amplicon size (bp), L. luteus CMS amplicon size (bp), amplification in other Lupin species (L. hispanicus), and the level of polymorphism on the L. luteus screening panel. [file 1471-2164-13-425-S1.pdf]

Lorena B Parra-Gonzalez, Gabriela A Aravena-Abarzúa, Cristell S Navarro-Navarro, Joshua Udall, Jeff Maughan, Louis M Peterson, Haroldo E Salvo-Garrido and Iván J Maureira-Butler. 2012. Yellow lupin (*Lupinus luteus*) transcriptome sequencing: molecular marker development and comparative studies. XX:xxx-xxx.

Supplemental table 2. Characteristics of 33 Conserved Microsynteny (CMS) markers developed in *L. luteus*. Shown for each primer pair are the Medicago chromosome library specificity, l1l2 isotigs where CMS forward and reverse primers were anchored, forward and reverse sequence, expected Medicago amplicon size (bp), *L. luteus* CMS amplicon size (bp), amplification in other Lupin species (*L. hispanicus*), and the level of polymorphism on the *L. luteus* screening panel

Name	Chr Mt	Isotig 1	Isotig 2	Forward primer (5'-3')	Reverse primer (5'-3')	Size *M. truncatula* (Kpb)	Size *L. luteus* (Kbp)	Amplification *L. hispanicus*	Polymorphism *L. luteus*
Synteny 1	3	l1l2itg51828	l1l2itg40136	CAAATCGCCGTAAACATCCG	ATTAGAATTGGCGGTGAAGGG	9.5	2.5	yes	
Synteny 2	3	l1l2itg46913	l1l2itg27310	AGCTTCCTAAAAGTGTTAGTGGACTTG	CTTCTTCACAATCTCATGCGG	11.0	1.35	yes	yes
Synteny 3	4	l1l2itg55311	l1l2itg53997	GGTATTCCCAAGCTCCTGCTC	GTTCGATCCACTTCATCAAGGC	6.1	2.2	yes	
Synteny 4	4	l1l2itg54281	l1l2itg43374	TGCCTTCAGTTTCCTATCATCTCC	TTCAGGGATGGTACATGTTCTTGC	0.7	0.8		
Synteny 5	1	l1l2itg48061	l1l2itg15278	GAGGGCATTGATGAGTTGATGG	TAGTAGAGGGCCGAATTCAAATGG	1.5	2.05	yes	yes
Synteny 6	4	l1l2itg55312	l1l2itg55312	CCCACATTCCACAGCCAAC	TGCCACTTCTTGGTGCCC	3.9	0.60		yes
Synteny 7	3	l1l2itg17143	l1l2itg29887	CTCAGTCAGATTCACACAGGACG	CCGCCTTAGGAGATAATCTACCC	1.7	1.30	yes	
Synteny 8	AC233577	l1l2itg12005	l1l2itg48071	GTTCTTTCCTCCCTTTCCCTTG	AATATTTTTAGCTGCCCATTTCTC	3.9	1.75	yes	yes
Synteny 9	6	l1l2itg05804	l1l2itg55313	CTCTACCGATGGTATGGACGAACC	GGAGATGATTCCGTACTTCAATTCG	1.0	1.2	yes	
Synteny 10	2	l1l2itg49108	l1l2itg54608	CAATCTGTTTTTCCAAATTCACACC	AAGGTAGAATAAAGGATGCTTCACG	0.5	0.7		
Synteny 11	2	l1l2itg51573	l1l2itg23123	ACATCCATCTGTTTTCAGGAATGC	GTGTGTGGAGAGGGAGTCATGC	1.7	2.05		yes
Synteny 12	AC233577	l1l2itg46168	l1l2itg12006	GTCAAGTGCCTAATTCATTGTGG	CGTGAAAGAGAGTAATCTCAACCC	13.5	1.3	yes	
Synteny 13	1	l1l2itg28004	l1l2itg54074	AAGGGATGATTTGGTCAACAGTGC	ACTTCTCTCCCCTCAGGGTTTAGG	1.0	1.80	yes	
Synteny 14	8	l1l2itg20287	l1l2itg41211	AATACCAAGTGCCCCATAAACC	TTCTGATACCCTGAACTATCTTCTCC	8.9	0.60		yes
Synteny 15	5	l1l2itg46890	l1l2itg46890	GCGTGGATCTCCGATTACC	TTTGCCAAAAGGGCTATTCC	6.9	1.70	yes	yes
Synteny 16	2	l1l2itg43843	l1l2itg56102	GGGAGAATAATTCCCGGTGAAG	GCCATAATAAGCCCAATAGGTCC	0.6	0.6	yes	
Synteny 17	5	l1l2itg55314	l1l2itg31802	CAAATATGTGCCGTTCTCATTTGG	AAAAGGAAGACTGATCGGTTCTGG	2.5	2.3	yes	
Synteny 18	7	l1l2itg13413	l1l2itg38851	AATCTCCTACACACATTGCTTGC	GGTCACCTTCTTGGTGCTTTG	3.7	0.5	yes	
Synteny 19	8	l1l2itg53474	l1l2itg30699	GACTTAATCCCAAATGTAACCAGC	ATCCATTTGCCCCATTCC	2.3	2.00	yes	
Synteny 20	5	l1l2itg47153	l1l2itg14477	CGAAAAATTGCTGCTGCATTACC	AATCATCAGGAAAGAGAGCCAAGG	1.9	3.5		
Synteny 21	5	l1l2itg25501	l1l2itg52471	TTCAGAACACACAACAAAACCATCC	AATCACCACCACCAGTTCTTGTCC	1.6	1.30	yes	yes
Synteny 22	5	l1l2itg29217	l1l2itg19759	TGATTCAGTCTCACTCCCAAACC	ATCTTCCTCCATTTCTCTTCCTACC	10.5	0.82	yes	yes
Synteny 23	7	l1l2itg38209	l1l2itg16703	TTCAGGGGAGGAAATTGTGTC	TGCTCGTACCTTATTGGGCTG	2.3	3.0	yes	
Synteny 24	1	l1l2itg38209	l1l2itg41211	TTGCTTGGTAAAGGCTATGCC	CGTGATGAGTGACAGTGCCTTC	2.2	3.0		
Synteny 25	5	l1l2itg42742	l1l2itg31773	GCTTACTTGCACTTGGTGGATGG	CTTTTGGGCAACATAGGAGAGAGG	1.3	2.8	yes	

Synteny 26	8	l1l2itg41211	l1l2itg43110	TTACTCCTTGCGGCGCTC	GGTGGCACTACAAGGCTTGAAG	9.3	1.3		
Synteny 27	3	l1l2itg33125	l1l2itg55315	GCTGCTCTAAAACTAGGTTTGTACTG	GTTTCCACTGCAGGAGTAACTAGG	6.4	0.8		
Synteny 28	3	l1l2itg41700	l1l2itg55316	AGGCACTTATAAAACACCATCTCC	GGAAGAAGCATCAGACTTGGTC	7.5	1.8		
Synteny 29	1	l1l2itg16703	l1l2itg38209	CTCGTACCTTATTGGGCTGACAG	TTCAGGGGAGGAAATTGTGTC	3.1	1.5	yes	
Synteny 30	7	l1l2itg16702	l1l2itg38209	GGGCCATGTGTTTCATTGC	TTGCTTGGTAAAGGCTATGCC	2.4	2.0	yes	
Synteny 31	AC235488	l1l2itg16601	l1l2itg49037	CAACACTCGTATTAATTTCCTCTTCG	TTTCTTATTTTCAGATCCTAGCGG	4.0	1.95	yes	
Synteny 32	1	l1l2itg26159	l1l2itg23875	AGATTCTTTTGGCACTGCTCTG	GTTGCATAACATGCTGCTTCTG	2.2	1.65	yes	yes
Synteny 33	3	l1l2itg55317	l1l2itg55318	TAAAGACGAAAAAGGAAAGCG	TGTAGTTAGTGGTTGTTTAGCAATGG	12.8	1.75	yes	
