# Supplementary material for: Yellow lupin (Lupinus luteus L.) transcriptome sequencing: molecular marker development and comparative studies
Source: BMC Genomics. 2012 Aug 24;13:425. doi: 10.1186/1471-2164-13-425 (PMC3472298; doi:10.1186/1471-2164-13-425)
Supplement: Additional file 2 — Table S2. Lupinus luteus, L. hispanicus and L. mutabilis accessions included in the study. [file 1471-2164-13-425-S2.pdf]

Lorena B Parra-Gonzalez, Gabriela A Aravena-Abarzúa, Cristell S Navarro-Navarro, Joshua Udall, Jeff Maughan, Louis M Peterson, Haroldo E Salvo-Garrido and Iván J Maureira-Butler. 2012. Yellow lupin (*Lupinus luteus*) transcriptome sequencing: molecular marker development and comparative studies. XX:xxx-xxx.

Supplemental table 1. *Lupinus luteus*, *L. hispanicus* and *L. mutabilis* accessions included in the study.

Accession name	Species	Country of origin	Seed source[a]
L2 (St.-332/55)	*L. luteus*	GER	W. K. Swiecicki, Institute of Plant Genetics
L10 (St.Treb.-10)	*L. luteus*	UKR	W. K. Swiecicki, Institute of Plant Genetics
L12 (Tomik)[b]	*L. luteus*	POL	W. K. Swiecicki, Institute of Plant Genetics
L13 (Jantar-stary)	*L. luteus*	POL	W. K. Swiecicki, Institute of Plant Genetics
L18 (Jantar)[b]	*L. luteus*	POL	W. K. Swiecicki, Institute of Plant Genetics
L 25(Dwarf mutant x STH 14-18)	*L. luteus*	POL	W. K. Swiecicki, Institute of Plant Genetics
L26 (St.Treb.-4151/61)	*L. luteus*	GER	W. K. Swiecicki, Institute of Plant Genetics
L 27 (St.Treb.-7)	*L. luteus*	POL	W. K. Swiecicki, Institute of Plant Genetics
L28 (St.-77)	*L. luteus*	GER	W. K. Swiecicki, Institute of Plant Genetics
L38 (St.-1/56)	*L. luteus*	GER	W. K. Swiecicki, Institute of Plant Genetics
L39 (Alteria)	*L. luteus*	GER	W. K. Swiecicki, Institute of Plant Genetics
L41 (Refusa)	*L. luteus*	GER	W. K. Swiecicki, Institute of Plant Genetics
L42 (St.Treb.-9)	*L. luteus*	GER	W. K. Swiecicki, Institute of Plant Genetics
L51 (St. Treb.-1332)	*L. luteus*	GER	W. K. Swiecicki, Institute of Plant Genetics
L53 (Mutant PW-5)	*L. luteus*	POL	W. K. Swiecicki, Institute of Plant Genetics
L54 (AFUS)	*L. luteus*	POL	W. K. Swiecicki, Institute of Plant Genetics
L55 (R-6040)	*L. luteus*	POL	W. K. Swiecicki, Institute of Plant Genetics
L56 (WTD-6142)	*L. luteus*	POL	W. K. Swiecicki, Institute of Plant Genetics
L59 (Manru)	*L. luteus*	POL	W. K. Swiecicki, Institute of Plant Genetics
L60 (Reda)	*L. luteus*	POL	W. K. Swiecicki, Institute of Plant Genetics
L67 (BSCh-621)	*L. luteus*	BYS	W. K. Swiecicki, Institute of Plant Genetics
L69 (BSCh-1033)	*L. luteus*	BYS	W. K. Swiecicki, Institute of Plant Genetics
L70 (BSCh-1064)[b]	*L. luteus*	BYS	W. K. Swiecicki, Institute of Plant Genetics
L71 (Poleszuk)	*L. luteus*	UKR	W. K. Swiecicki, Institute of Plant Genetics
L73 (Ryast)[b]	*L. luteus*	UKR	W. K. Swiecicki, Institute of Plant Genetics
L75 (WTD 6179)	*L. luteus*	POL	W. K. Swiecicki, Institute of Plant Genetics
L76 (Borsaja)	*L. luteus*	GER	W. K. Swiecicki, Institute of Plant Genetics
L77 (Borselfa)	*L. luteus*	GER	W. K. Swiecicki, Institute of Plant Genetics
L81 (Naroczanskij)	*L. luteus*	BYS	W. K. Swiecicki, Institute of Plant Genetics
L84 (Kroton)	*L. luteus*	POL	W. K. Swiecicki, Institute of Plant Genetics
L85 (Wodjil)	*L. luteus*	AUS	W. K. Swiecicki, Institute of Plant Genetics
L87 (Markiz)	*L. luteus*	POL	W. K. Swiecicki, Institute of Plant Genetics
L88 (Juno)	*L. luteus*	POL	W. K. Swiecicki, Institute of Plant Genetics
L 91 (PI505853)	*L. luteus*	RUS	Western Regional PI Station, USDA
L92 (PI 505854)	*L. luteus*	RUS	Western Regional PI Station, USDA
L100 (PI 505850)	*L. luteus*	RUS	Western Regional PI Station, USDA
L103 (PI 384565)	*L. luteus*	POL	Western Regional PI Station, USDA

L104 (PI 384568)[b]	*L. luteus*	PORT	Western Regional PI Station, USDA
L106 (PI 289168)	*L. luteus*	HUNG	Western Regional PI Station, USDA
L108 (PI 368919)	*L. luteus*	HUNG	Western Regional PI Station, USDA
L111 (PI505855)	*L. luteus*	GER	Western Regional PI Station, USDA
L112 (PI516633)	*L. luteus*	GER	Western Regional PI Station, USDA
L116 (PI 516635)	*L. luteus*	MORO	Western Regional PI Station, USDA
L117 (PI 516635)	*L. luteus*	MORO	Western Regional PI Station, USDA
L120 (PI533712)[b]	*L. luteus*	SPN	Western Regional PI Station, USDA
L122 (PI505856)	*L. luteus*	POL	Western Regional PI Station, USDA
L123 (PI316285)	*L. luteus*	ISRL	Western Regional PI Station, USDA
L 124 (PI316284)	*L. luteus*	PORT	Western Regional PI Station, USDA
L125 (270GY-215)	*L. luteus*	CHIL	CGNA
L126 (270GY-226)	*L. luteus*	CHIL	CGNA
L129 (270GY-247)	*L. luteus*	CHIL	CGNA
L192 (WTD 6121)	*L. luteus*	POL	W. K. Swiecicki, Institute of Plant Genetics
L193 (Leniniec)	*L. luteus*	UKR	W. K. Swiecicki, Institute of Plant Genetics
L195 (P1-HS)	*L. luteus*	CHIL	CGNA
L204 (716-HS)	*L. luteus*	CHIL	CGNA
L206 (771-HS)	*L. luteus*	CHIL	CGNA
L207 (775-HS)	*L. luteus*	CHIL	CGNA
L208 (790-HS)	*L. luteus*	CHIL	CGNA
L213 (mic1)	*L. luteus*	CHIL	CGNA
L220 (mic70)	*L. luteus*	CHIL	CGNA
L221(mic74)	*L. luteus*	CHIL	CGNA
L222 (mic189)	*L. luteus*	CHIL	CGNA
L223 (mic423)	*L. luteus*	CHIL	CGNA
L139 (PI 385136)	*L. hispanicus*	SPN	Western Regional PI Station, USDA
L174 (PI457964)	*L. mutabilis*	PER	Western Regional PI Station, USDA

[a]Complete Seed source addresses: (i) Dr. W. K. Swiecicki, Institute of Plant Genetics, Polish Academy of Sciences, Poznan, Poland; (ii) Western Regional PI Station, USDA, ARS, WRPIS, Washington State University, Regional Plant Introduction Station, Pullman, Washington, USA; and (iii) Agri Aquaculture Nutritional Genomic Center (CGNA), Plant Biotechnology Unit INIA-Carillanca, Camino Vilcún Km 10, Temuco, P.O Box 58-D, Chile. (RUS: Russia, ISRL: Israel, HUNG: Hungary, CHIL: Chile, GER: Germany, SPN: Spain, PORT: Portugal, MORO: Morocco, POL: Poland, BYS: Belarus, UKR: Ukraine, PER: Peru).

[b]Diverse genotypes included in the screening panel.
